# Detection Accuracy of [ ^68^ Ga] PSMA PET/CT with Rising PSA in Prostate Cancer

**DOI:** 10.1055/s-0045-1804894

**Published:** 2025-02-27

**Authors:** Parul Mohan, Palak Wadhwa, Harsh Mahajan, Dileep Kumar, Giacomo Aringhieri, Dania Cioni

**Affiliations:** 1 Department of Nuclear Medicine, Mahajan Imaging and Labs, New Delhi, India; 2 Central Research Institute, Shanghai United Imaging Healthcare, Shanghai, China; 3 Department of Nuclear Medicine & PET/CT, University of Pisa, Pisa, Italy

**Keywords:** PSA, PSMA PET/CT, digital PET/CT scanner, prostate cancer, prostate-specific antigen, molecular imaging, biomarkers

## Abstract

**Objective** The objective of this study was to evaluate the clinical utility of gallium-68 [ ^68^ Ga] prostate-specific membrane antigen (PSMA) positron emission tomography/computed tomography (PET/CT) with rising prostate-specific antigen (PSA) levels in prostate cancer diagnosis.

**Methods** This is a retrospective, single-center, observational cross-sectional study, which is provided after ethics committee clearance, from May 2, 2022 to June 25, 2022. Study includes sample size of 50 patients with prostate adenocarcinoma with varying PSA levels and Gleason score of 6 to 9 who underwent [ ^68^ Ga] PSMA PET/CT scan. The patients included in this study underwent PET/CT scan on uMI550 (United Imaging Healthcare, Shanghai, China).

**Results** All patients were divided into three groups based on PSA levels in ng/mL as: PSA ≤ 0.2 (8%), 0.2 < PSA ≤ 1 (10%), 1 < PSA ≤ 3 (8%), 3 < PSA ≤ 10 (18%), and PSA > 10 (56%). Among 50 scans, at least one PSMA avid lesion was visualized in 41 scans (78.9%). These scans were considered positive and included in this study, rest of the scans had insignificant PSMA uptake and were considered negative. [ ^68^ Ga] PSMA PET/CT detection rates were 75.0, 20.0, 50.0, 88.90, and 89.3% in patients with PSA ≤ 0.2, 0.2 < PSA ≤ 1, 1 < PSA ≤ 3, 3 < PSA ≤ 10, and PSA > 10, respectively. In addition to prostate bed, lesions were also visualized in lymph nodes (32%), liver (2%), skeleton (28%), and thorax (6%). Considering lesions in the prostate bed a significant direct correlation was detected between maximal standardized uptake value (SUVmax) and PSA value ( *p* = 0.03).

**Discussion** PSMA PET/CT has been demonstrated to be an effective method for identifying both low-grade Gleason score tumors and low PSA levels. The study provides support for the use of [ ^68^ Ga] PSMA PET/CT in conjunction with PSA levels for the evaluation of prostate cancer, including local recurrence and distant metastases.

**Conclusion** The findings of this study indicate that PSMA PET/CT is an effective method for diagnosing prostate cancer, as it allows for the detection of high SUVmax values in pathological tissues. Furthermore, high sensitivity and detection rates are noted with PSMA PET/CT scan even in cases where PSA levels were low. Therefore, this study demonstrates that [ ^68^ Ga] PSMA PET/CT is beneficial for the early detection of prostate cancer and the prediction of treatment outcomes.

## Introduction

Prostate cancer is one of the primary health concerns because, among the worldwide male population, it is the fifth most prevalent cause of cancer-related deaths and the second most common male malignancy. 1 2 3 4 5 As the optimal management of prostate cancer relies on accurate diagnosis, staging, and assessment of treatment response, these fields are constantly developing.

In the last few years, improvements in molecular imaging and biomarker development have profoundly changed the field of prostate cancer diagnostics. 6 Prostate-specific membrane antigen (PSMA) positron emission tomography (PET)/computed tomography (CT) imaging and prostate-specific antigen (PSA) are those improvements, which became essential tools for comprehensive evaluation and personalized management. 7 8

PSMA PET/CT is an innovative hybrid imaging technique that combines the information about metabolic activity and function of tissues and organs obtained from PET with the precise anatomical details provided by CT scans. PSMA is a transmembrane protein that is highly expressed on the surface of prostate cancer cells, representing an optimal target both for imaging and therapy. 9 PSMA-targeted radiotracers, labeled with radioactive isotopes, as gallium-68 [ ^68^ Ga], selectively bind to PSMA receptors, enabling the visualization of prostate cancer lesions. 10 Fusion of PET and CT images offers superior sensitivity and specificity in detecting prostate cancer lesions, particularly in cases of biochemical recurrence and metastatic disease. 11

PSA is an enzyme, serine protease, produced by the prostate gland, which is already proven and used for a long time as a biomarker for prostate cancer screening, diagnosis, and treatment monitoring. 12 13 14 PSA levels in blood reflect prostate gland activity, and alterations in PSA levels may indicate the presence of prostate cancer. PSA screening enables the early detection of prostate cancer, as it is the most common initial laboratory abnormality in the absence of symptoms, which allows timely interventions and improved outcomes. 15

Although PSA is a highly sensitive marker, it is also relatively nonspecific and inaccurate as a screening tool, because both benign and malignant processes lead to an increase in serum levels of this marker. 16 This underscores the importance for the development of new molecular markers and further investigations in this field to enhance cancer detection and management.

In this article, we endeavor to study the role of [ ^68^ Ga] PSMA PET/CT in the diagnostics of prostate cancer and management. Additionally, this article focused on establishing a correlation between [ ^68^ Ga] PSMA PET/CT and PSA levels and Gleason score. Additionally, this article will also illustrate the normal uptake patterns of [ ^68^ Ga] PSMA PET/CT in healthy individuals and pathological or metastatic uptake in diseased individuals.

## Methods and Materials

### Place of Study

The study was conducted in the Department of Nuclear Medicine and PET/CT, Mahajan Imaging & Labs, Safdarjung Development Area, New Delhi, India.

### Study Type

This study was a retrospective, single-center, observational cross-sectional study.

### Time Period

After ethics committee clearance, the study was conducted from May 2, 2022 to June 25, 2022.

### Study Population

All consecutive patients detected with adenocarcinoma prostate (mean age ± standard deviation, 71.0 ± 9.1 years) with varying PSA levels and Gleason score of 6 to 9 who underwent [ ^68^ Ga] PSMA PET/CT scan at our department.

### Inclusion Criteria

- Patients who give consent.

### Exclusion Criteria

- Patients having prostate carcinoma as second primary or synchronous primary (dual malignancy).

## Methodology

### Study Method

1. Prior permission and approval from the protocol and ethics committee.
2. The prospective study was undertaken in carcinoma prostate patients, who were referred to our department for [ ^68^ Ga] PSMA PET/CT scan as part of their staging workup.
3. After applying inclusion and exclusion criteria, the patients were enrolled for the study.
4. An informed written consent to participate in the study was taken from all the enrolled patients ( **Appendix II** and **III** ).
5. Demographic data of each patient was recorded, including relevant history, clinical examination findings, and findings of conventional radiological imaging in the pro forma attached herewith ( **Appendix I** ).

### [ ^68^ Ga] PSMA PET/CT Scan Procedure

All the patients were given intravenous injection of 132 to 222 MBq (4–6 mCi) of [ ^68^ Ga] PSMA followed by PET/CT scanning. The scanning was done from the vertex to the mid-thigh using a dedicated digital PET/CT scanner (uMI550, United Imaging Healthcare) with 24 cm of axial field of view PET component having a high spatial resolution and sensitivity, and a 80-slice CT system with 0.5 second rotation within 45 ± 15 minutes of injecting [ ^68^ Ga] PSMA intravenously.

A whole-body diagnostic CT scanning was performed first (120 kV, 200 mA, 0.8 seconds per CT rotation, pitch of 1.375:1, and table speed of 27.55 mm/sec), 0.55 mm slice thickness with reconstruction interval of 1.0 mm with standard reconstruction kernel with additional breath hold CT for evaluation of the lungs. PET scanning was performed immediately after acquisition of the CT images, without changing the patient position. Imaging was performed with five and eight bed positions, an acquisition time of 2 minutes for each bed position, a 15% overlap in 15.7 cm axial field of view, and 192 * 192 reconstruction matrix. The emission data was attenuation corrected along with scattering, random, and decay correction. A delayed sequence of pelvis was acquired after furosemide injection.

### PET/CT Image Interpretation

Images were interpreted in advanced PET/CT software (uWS PET/CT, UIH with uAI advanced reconstruction algorithms) workstation equipped with fusion software that enables the display of PET images with and without attenuation correction, CT images, and fused PET/CT images. Reconstruction was conducted with an ordered subset expectation maximization (OSEM) and HYPER deep progressive reconstruction (DPR, which is the only artificial intelligence-based PET reconstruction technology that is trained on high-count and total-body PET data to support small lesion detectability and improve quantitative accuracy) algorithm incorporated with the point spread function to maintain the uniform resolution across the field of view. 17 18 19 The OSEM and HYPER DPR reconstruction was performed using two iterations/18 subsets and Gaussian-filtered to the spatial resolution of less than 3 mm at full width at half maximum. Attenuation correction was performed with contrast-enhanced CT data with automatic contrast correction algorithm. The PET and CT data were acquired with single contrast injection and single CT acquisition for both attenuation correction and coregistration in PET/CT protocol on uMI 550 scanner. Due to high contrast and significantly higher accumulation of [ ^68^ Ga] PSMA in the malignant tissue, 1 hour delayed images do not affect the assessment of prostatic bed even though there is urinary bladder activity. Dual-phase scan for the prostate bed is not necessary unlike ^18^ F/ ^11^ C-choline scans in our experience.

All scans were evaluated independently and blindly by two experienced nuclear medicine physicians. PET images were looked for area of increased radiotracer uptake. Uptake sites were interpreted on the basis of shape, location, and intensity. Each lesion was assessed on transverse, coronal, sagittal, and three-dimensional maximum intensity projection images and its [ ^68^ Ga] PSMA uptake was expressed as the maximal standardized uptake value (SUVmax) corrected for the administered dose and patient body weight.

All PSMA avid foci with abnormal tracer uptake that could not be explained by physiological [ ^68^ Ga] PSMA activity were labeled as pathological and considered as malignant. This is true due to substantially high sensitivity of [ ^68^ Ga] PSMA radiotracer. A finding was considered equivocal in the presence of urinary activity in a typical anatomic location or when a lesion with an abnormal tracer accumulation showed mild intensity and had no definite morphologic findings. 20 The findings on delayed acquisition were used to better classify such lesions as benign or malignant. PSMA avid uptake was visible in anatomical locations including the prostate bed, lymph nodes (including para-aortic, mesenteric, aortocaval, iliac, pararectal, and other pelvic lymph nodes), skeleton, liver, and thorax.

A SUVmax value of 4.0 was set as the cutoff value. Thus, SUVmax values of 4.0 and above in the regions of interest (ROIs) drawn over the prostatic bed (primary or recurrence) were considered as suspicious of malignancy or recurrent tumor. 21 Any abnormal [ ^68^ Ga] PSMA scan outside the prostate bed especially in lymph nodes and skeleton and in any visceral organs was considered as highly probable for metastatic disease. For calculation of the SUVmax, ROIs were highlighted with cursor selection around areas with focally increased uptake in transaxial slices and automatically adapted to a three-dimensional volume of interest at a 42% threshold segmentation method. The maximum of all SUVmax was recorded for each anatomical location.

## Results

### PSMA Detection Rate

The patients were categorized into three groups based on PSA levels in ng/mL as: PSA ≤ 0.2 (8%), 0.2 < PSA ≤ 1 (10%), 1 < PSA ≤ 3 (8%), 3 < PSA ≤ 10 (18%), and PSA > 10 (56%). Positive scans were considered where at least one PSMA avid lesion was visualized and there were 41/50 (78.9%) positive scans included in this study. Negative scans were considered where there was insignificant PSMA uptake.

**Fig. 1:**
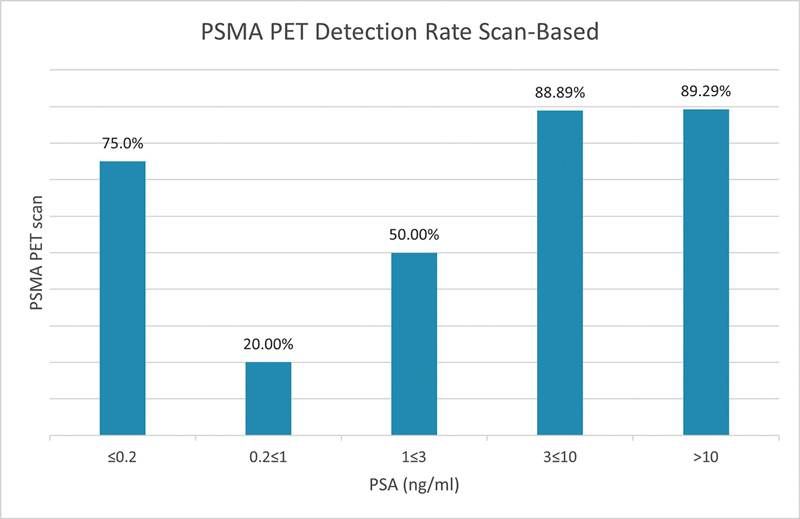
Comparative analysis between prostate-specific membrane antigen positron emission tomography/computed tomography ([ ^68^ Ga] PSMA PET/CT) detection rates and prostate-specific antigen (PSA) levels of ≤ 0.2 ng/mL, 0.2–1 ng/mL, 1–3 ng/mL, 3–10 ng/mL, and > 10 ng/mL.

[ ^68^ Ga] PSMA PET/CT detection rate is demonstrated in Fig. 1 . The detection rates were 75.0, 20.0, 50.0, 88.90, and 89.3% in patients with PSA ≤ 0.2, 0.2 < PSA ≤ 1, 1 < PSA ≤ 3, 3 < PSA ≤ 10, and PSA > 10, respectively.

There was a significant correlation between [ ^68^ Ga] PSMA PET/CT positivity and PSA >3 ( *p* = 0.02) as well as Gleason score ( *p* = 0.002). However, there was no significant correlation between [ ^68^ Ga] PSMA PET/CT positivity and primary clinical parameters such as initial PSA < 3.

Apart from prostate bed, lesions were also visualized in lymph nodes (32%) out of which 10% are in the head and neck, liver (2%), skeleton (28%), and thorax (6%).

### SUVmax versus PSA

**Fig. 2:**
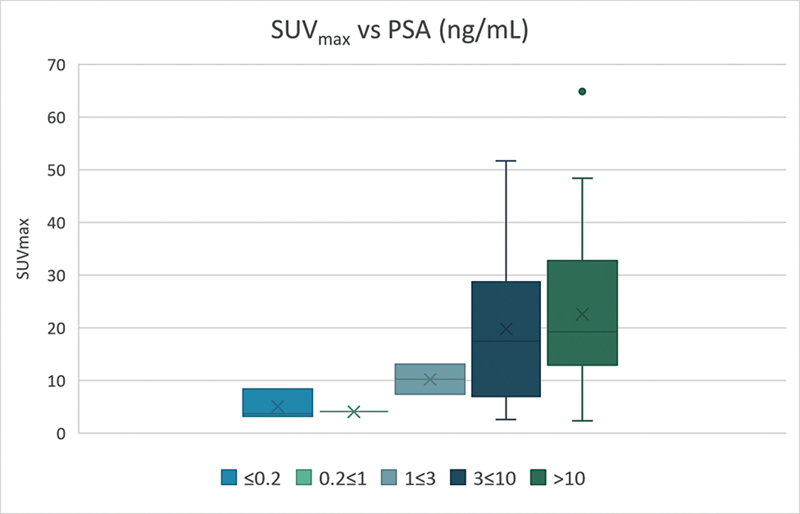
Correlation between the maximal standardized uptake value (SUVmax) of prostate-specific membrane antigen positron emission tomography/computed tomography ([ ^68^ Ga] PSMA PET/CT) for 41 positive scans and prostate-specific antigen (PSA) levels. The SUVmax used here was the maximum of all the detected lesions in the prostate bed.

There was a significant correlation between SUVmax and PSA value for lesions in prostate bed ( *p* = 0.03). With higher PSA values, there was an increase in SUVmax as can be seen in Fig. 2 . The greatest SUVmax was 64.9 at a PSA level of 22.

### Normal Biodistribution of PSMA

**Fig. 3:**
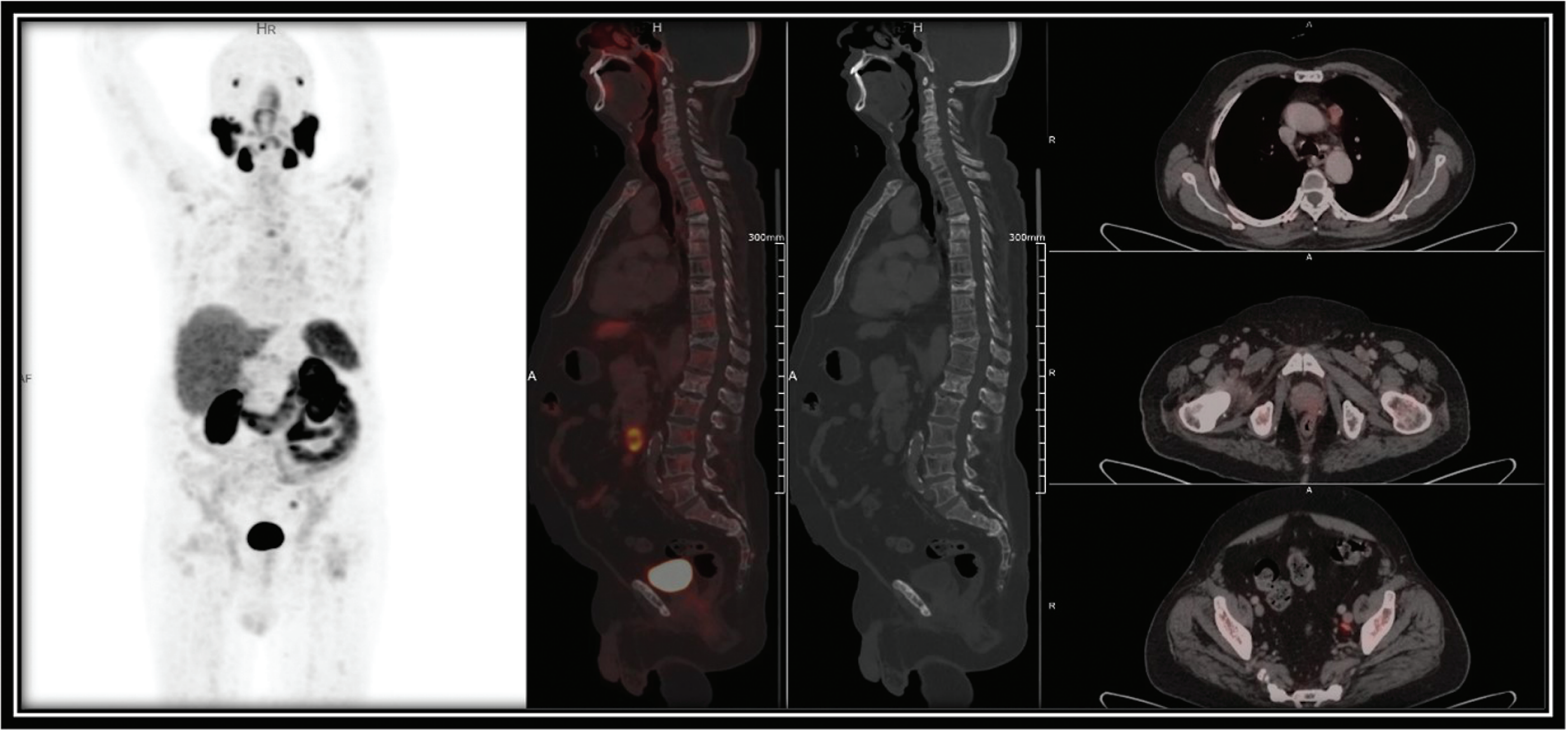
Prostate-specific membrane antigen positron emission tomography/computed tomography ([ ^68^ Ga] PSMA PET/CT) images show absence of PSMA avid visible mitotic disease in the body.

An 80-year-old male, recently diagnosed with adenocarcinoma prostate gland (Gleason's score 4 + 4 = 8) and raised total serum PSA level of approximately 11.7 ng/mL, underwent [ ^68^ Ga] PSMA PET/CT scan for pretreatment staging, as shown in Fig. 3 , demonstrated normal PSMA biodistribution and an absence of PSMA avid visible mitotic disease in the body.

**Fig. 4:**
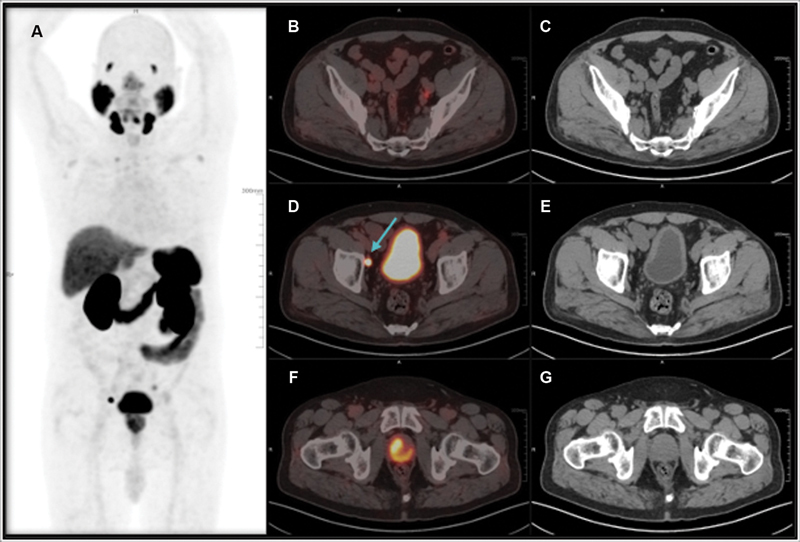
Prostate-specific membrane antigen positron emission tomography/computed tomography ([ ^68^ Ga] PSMA PET/CT) images show the maximum intensity projection of [ ^68^ Ga] PSMA PET/CT demonstrating ill defined primary and lymph node lesions ( **A** ) PSMA avid ill-defined lesion in the prostate gland ( **B** , **D** ) and PSMA avid bilateral external iliac lymph nodes ( **B** – **E** ).

Another case of 64 years old male, recently diagnosed with acinar adenocarcinoma prostate gland (Gleason's score 3 + 4 = 7) and raised total serum PSA level of approximately 8.2 ng/mL, underwent [ ^68^ Ga] PSMA PET/CT scan for pretreatment staging is shown in Fig. 4 . As can be seen in Fig. 4 , [ ^68^ Ga] PSMA PET/CT images show PSMA avid ill-defined lesion in the prostate gland ( Fig. 4B, D ) and PSMA avid bilateral external iliac lymph nodes ( Fig. 4B–E ) apart from normal biodistribution of PSMA.

### Metastatic Image of PSMA

**Fig. 5:**
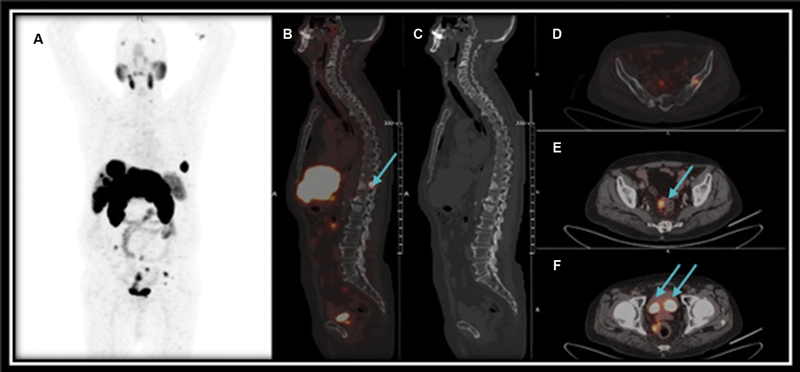
Prostate-specific membrane antigen positron emission tomography/computed tomography ([ ^68^ Ga] PSMA PET/CT) images show PSMA avid ill-defined lesions in the prostate gland, multiple PSMA avid abdomino-pelvis lymph nodes with multiple PSMA avid liver lesions, and multiple PSMA avid and non-PSMA avid skeletal lesions, likely metastatic disease.

A 77-year-old male, follow-up case of adenocarcinoma prostate gland, postmultiple cycles of chemotherapy and radiotherapy and raised total serum PSA level of approximately 152.4 ng/mL, underwent [ ^68^ Ga] PSMA PET/CT scan for treatment response evaluation and restaging, shown in Fig. 5 , demonstrated multiple PSMA avid abdomino-pelvis lymph nodes with multiple PSMA avid liver lesions and multiple PSMA avid and non-PSMA avid skeletal lesions, likely metastatic disease

## Discussion

Our retrospective study had following objectives: (1) correlating the PSA with (i) [ ^68^ Ga] PSMA PET/CT detection rates and (ii) SUVmax, and (2) demonstrating the normal uptake patterns of [ ^68^ Ga] PSMA PET/CT in healthy individuals and abnormal lesion or metastatic uptake in diseased individuals.

Previous studies showed that [ ^68^ Ga] PSMA PET/CT represents promising radiotracer that enables staging and management of prostate cancer. 22 23 24 Earlier research has also demonstrated to have close correlation between PSA values and [ ^68^ Ga] PSMA PET/CT positivity for higher PSA values. However, this was not the same for lower PSA values and the correlation between [ ^68^ Ga] PSMA PET/CT diagnostic accuracy and low PSA values is still unclear. Further, a retrospective study with large patient population including 1,007 prostate cancer patients have shown an overall detection rate of 79.5% along with the sensitivities of 46, 46, and 73% for PSA levels of 0.2 ng/mL or less, 0.21 to 0.5 ng/mL, and 0.51 to 1.0 ng/mL, respectively. 22 25 26 The results of this study are in accordance with those of previous studies indicating an overall detection rate of 78.9% along with the sensitivities of 75.0, 20.0, 50.0, 88.90, and 89.3% in the patients with PSA ≤ 0.2, 0.2 < PSA ≤ 1, 1 < PSA ≤ 3, 3 < PSA ≤ 10, and PSA > 10, respectively. We observe a higher detection rate for even very low PSA level of less than and equal to 0.2 (please try to explain or hypothesize why). This demonstrates that PSMA PET is highly sensitive in the detection of prostate cancer, and it could be very useful for the detection of small metastases, localized disease with small foci of cancer cells, and other cases where PSA levels are low. Since prostate cancer is a heterogeneous disease, there could be regions with high concentration of cancer cells in prostate whereas overall PSA levels could be low.

Our study also reveals a significant correlation between [ ^68^ Ga] PSMA PET/CT positivity and PSA > 3 ( *p* = 0.02) as well as Gleason score ( *p* = 0.002), which corresponds with previous studies, whereas the same was not observed for PSA < 3. Lesions were also visualized in lymph nodes (32%) out of which 10% were in the head and neck, liver (2%), skeleton (28%), and thorax (6%). Furthermore, a notable direct correlation was identified between SUVmax and elevated PSA levels.

Interestingly, that low Gleason score and low PSA levels do not exclude cancer, as [ ^68^ Ga] PSMA scans showed high radiotracer accumulation in such cases. A PSA of 3.0 ng/mL was found to have a sensitivity of over 80%, which implies that 20% of cancers are missed when only the PSA level is obtained. A biopsy or follow-up imaging will show the true validity of Ga68-PSMA scans. 27 This is the reason why in our data we could not arrive at a meaningful correlation of Ga68-PSMA scan with biochemical response.

The sensitivity observed in this study is in correspondence to that reported in the previous studies. 28 29 30 As most of our scans were done on patients with histopathologically proven prostate cancer, we had small number of benign and noncancerous prostate lesions, which rendered an assessment of specificity of [ ^68^ Ga] PSMA scans impossible.

Additionally, sensitivity for distant prostate metastasis was also high with small lesions being detected in the skeleton and lymph nodes even with low PSA levels.

Hence, we postulate that [ ^68^ Ga] PSMA scan should be used for the evaluation of local recurrence, any distant metastases, and response to cancer-directed therapy in prostate cancer along with PSA values. PSA values ranged from 0.008 to 766.7 ng/mL. In one case with low PSA values of 0.005, the patient had mild PSMA avid fibroatelectatic lesion in the lower lobe of the right lung with perilesional ground-glass opacities and interstitial thickening. Diffuse ground-glass haziness is also noted in the rest of bilateral lung fields.

It is known that PSMA is a cell surface protein that is expressed at higher levels in the prostate carcinoma cells, in contrary to other PSMA-expressing tissues that express PSMA at normal levels. Among them low levels of PSMA is expressed in salivary glands and kidney. Normal levels of PSMA are expressed in tissues including the lacrimal glands, liver, spleen, duodenum, colon, and prostate. Our data correlates with the literature and our imaging analysis has indicated normal biodistribution of [ ^68^ Ga] PSMA in an 80-year-old male with the absence of PSMA avid mitotic disease. Fig. 3 illustrated that normal level of uptake is observed in the salivary glands, lacrimal glands, kidney, spleen, liver, bowel, and prostate.

This study additionally illustrated ill-defined lesions in the prostate and iliac lymph nodes as shown in Fig. 4 . This highlights that metastatic disease usually has excellent contrast and high PSMA uptake in the lesions. In addition, the SUVmax was high for metastatic disease even for low PSA levels. It was also demonstrated that PSMA is a highly specific radiotracer for detection, staging, and disease management of prostate carcinoma as can be seen in Fig. 5 .

Through the years, PSA has provided significant advancements in the diagnosis and prognosis of prostate cancer, although it was counterbalanced by its low sensitivity and specificity. Recently, PSA has been thrust into the public spotlight after several publications showed discrepancy in PSA level and burden of cancer. Thompson et al reported that 15% of men with a PSA value less than 4.0 ng/mL, the cutoff value for potential biopsy, were found to have cancer. 31 To increase the accuracy and prediction, PSA kinetics, including PSA density and PSA velocity (PSAV), have been proposed.

There is a need to develop novel molecular markers (PCA3 or molecular markers, i.e., cell cycling processing genes) and we highly encourage more future well-designed prospective studies employing the standard definition and calculation of PSAV.

## Conclusion

The results of this study show the clinical utility of [ ^68^ Ga] PSMA PET/CT in prostate cancer and present the biodistribution of [ ^68^ Ga] PSMA PET/CT in normal and diseased cases. In healthy individuals, normal level of uptake is observed in the salivary glands, lacrimal glands, kidney, spleen, liver, bowel, and prostate, whereas in diseased individuals, well- and ill-defined lesions are detected with high SUVmax values. This article also suggests that [ ^68^ Ga] PSMA scan had high sensitivity and detection rates with even low PSA levels. Further, this study shows that [ ^68^ Ga] PSMA PET/CT is helpful in the early detection of prostate cancer and the prediction of the treatment.

## Notes

Appendix I

Patient Pro forma
Serial no: _______ Date:______________
Referring Consultant: _______________________________
(A) Particulars of the patient :
  1. Name: Mr. /Ms.: ________________________________
  2. Age: _______ years.
  3. Sex: _____________
  4. Residential address: ________________________________
  5. Phone no: ________________________________
  6. E mail ID:_____________________
(B) History of the patient :
  (i) Present complaints: __________________________________
  (ii) History of Past illness:
    - **Diabetes mellitus: Yes _______________No___________**
    - **Hypertension: Yes _______________No___________**
    - **Past History of Surgery: Yes_______________ No____________**
  (iii) Family history: __________________________________________
  (iv) Treatment history: _________________________________________
  _____________________________________________________________
(C) Investigations:
  1. Hematology: Hemoglobin ___________mg/dl
    Total leukocyte count ____________/cubic mm
    Differential leukocyte count____________
  2. **Biochemistry:** Random blood sugar ________mg/dl
    Serum creatinine ___________mg/dl
    Serum urea _________________mg/dl
    Serum PSA—i) Total_________ng/ml ii)Free_________ng/ml
    Urinalysis_______________________________
  3. **Histopathology:** Gleason score _____________________
    Gleason Grade _____________________
  4. **CT Scan** ________________________________________
  5. **MRI** ________________________________________
  6. **Any other relevant investigation:** ________________________________________________________________________________________________________________
  7. Findings on ^68^ Ga-PSMA PET/CT scan:

## Notes

Appendix II
**Patient Information Sheet**
Dear Sir/Madam,
You are invited to participate in a study entitled **“ .”** The study will be conducted in the department of Nuclear Medicine & PET/CT, Mahajan Imaging and Labs New Delhi, India. The information in this sheet will help you to decide whether or not to participate in the proposed study. You will be one of the participants we plan to recruit in this study.

**Procedure**

If enrolled and consented, you will undergo a Positron Emission Tomography (PET) scan, using a radio-labeled tracer called ^68^ Ga PSMA. PET-CT scan combines both a Computerized Tomography (CT) scan and a PET scan done on the same machine during one procedure. For the purposes of the rest of this consent form, we will refer to this combined scan as ^68^ Ga PSMA PET scan.
You will have an IV catheter inserted. At the same time, a small amount of blood will be taken to measure your blood glucose level.
You will receive an IV dose of ^68^ Ga PSMA in a dose determined by your weight. The injection volume will be very small and it will only take a few seconds to administer through your IV.

You will rest in a private quiet room for 45 to 60 minutes, to enable the ^68^ Ga PSMA to circulate throughout your body.

You will undergo the ^68^ Ga PSMA scan, which will take ∼30 minutes. You will need to lie still on the scanner bed. During the scan, the staff will monitor you by direct vision.

**After the Procedure**

After the scan is completed, you are free to leave the center and will be encouraged to drink 3 to 4 extra glasses of water by the end of the day to promote further clearance of the remaining ^68^ Ga PSMA tracer from your body.

**Safety Profile of the Method**
^68^ Ga PSMA PET scans are considered very safe procedure and pose no extra risk or suffering. There have never been any reported side effects attributed to the ^68^ Ga PSMA tracer. There is no published data that any side effects in humans have ever been shown to be directly related to radiation exposure subjects have received from ^68^ Ga PSMA PET scans. You should not have any complications in the future as a result of the ^68^ Ga PSMA scan.

**Confidentiality and Privacy of Records**
The data generated from your participation will be saved and maintained with due confidentiality and privacy by investigators and only authorized persons will be allowed to see them. You have the right to confidentiality regarding the privacy of your medical information (personal details, results of physical examinations, investigations, and your medical history). By signing this document, you will be allowing the research team investigators, other study personal, institutional ethics committee and any other person or agency required by law to view your data, if required. The information from this study, if published in scientific journals or presented at scientific meetings, will not reveal your identity.
**Benefit of Participation**
Indirectly your PSMA PET scan may help your doctor to offer more appropriate treatment for you. Your participation in this study may allow the investigators to gain valuable knowledge that may benefit others in the future.
**Risk of Participation**
The participation in the proposed study is not expected to evoke any undue risk or side effects.
**Finances Involved**
The participation in the project will not add/incur extraordinary expenditure on the patient.
**Number of Participants**
We plan to recruit 50 patients for the study.
**Freedom to Participate and Withdraw from the Study**
You shall be required to sign a form for informed consent to participate in the research work, which shall be signed by the treating doctor as well. You would be required to sign a copy of information sheet after writing “I have been given a copy of information sheet” and return it back to the treating doctor. You should feel free to ask any questions about the research work and the treating doctor shall be willing to answer them. If at any moment you decide to withdraw from the study, you shall be free to do so. By doing so, you will not experience any loss.

## Notes

Appendix III
**Informed Consent Form**

STUDY TITLE:
“ ”
Regd. Number: ____________________________________________________________
Patient Name: _____________________________________________________________
Sex (Male/Female):__________________________________________________________
Age (Years):________________________________________________________________
Date (DD/MM/YYYY): ______________________________________________________

**Informed Consent**
(Permission to participate in Research)

1. The contents of information sheet dated..………………that was provided, have been read carefully to me/ explained to me, in language that I comprehend and I have fully understood the contents.
2. I confirm that I have read and understood the information sheet for the above study and have had the opportunity to ask questions.
3. I understand that my participation in this study is voluntary and that I am free to withdraw at any time, without giving any reason, without my medical care or legal rights being affected.
4. I agree not to restrict the use of any data or results that arise from this study provided such a use is only for scientific purposes.
5. I understand that my treatment will not be affected if I refuse to participate. I have informed that I can discontinue my participation at any time.
6. My identity will be kept confidential. I agree that I will not demand any limits on the use of the findings of the study.
7. I authorize the assess to my medical records as explained in this information and consent form.
8. I agree with my free and full consent to participate in the study.

Name of the patient-Participant: Signature…………………………………Date……………………. Place …………………. Name of the Witness Signature…………………………………Date……………………. Place ………………….
